# Least-detectable and age-related local *in vivo* bone remodelling assessed by time-lapse HR-pQCT

**DOI:** 10.1371/journal.pone.0191369

**Published:** 2018-01-24

**Authors:** Patrik Christen, Stephanie Boutroy, Rafaa Ellouz, Roland Chapurlat, Bert van Rietbergen

**Affiliations:** 1 Institute for Biomechanics, ETH Zurich, Zurich, Switzerland; 2 Orthopaedic Biomechanics, Department of Biomedical Engineering, Eindhoven University of Technology, Eindhoven, The Netherlands; 3 INSERM UMR 1033, Université de Lyon, Lyon, France; University of Notre Dame, UNITED STATES

## Abstract

We previously developed an image analysis approach for the determination of local sites of bone remodelling using time-lapse *in vivo* HR-pQCT. The involved image filtering for removing noise was chosen rather aggressively, and also removed some effects of the bone remodelling. In this paper, we quantify these filtering settings using *ex vivo* reproducibility HR-pQCT images, and determine the least-detectable bone remodelling using *in vivo* reproducibility HR-pQCT images, as well as testing whether the approach is capable of capturing age-related bone remodelling by use of *in vivo* long-term HR-pQCT images. We found that a threshold value of 225 mg HA/cm^3^ for the filtering led to acceptable results with falsely determined bone remodelling of less than 0.5%, and that the least-detectable bone formation and bone resorption are 2.0 ± 1.0% and 2.2 ± 0.7% respectively. We also found that age-related local bone remodelling can be captured satisfactorily in postmenopausal women. The latter revealed new insights into the effect of ageing on bone remodelling, and showed that bone remodelling seems to take place through a few small formation packets and many large resorption volumes leading to a net bone loss. We conclude that local *in vivo* bone remodelling can be successfully assessed with time-lapse *in vivo* HR-pQCT capable of assessing age-related changes in bone remodelling.

## Introduction

Bone remodelling is a tightly coupled mechanism consisting of repetitive cycles of bone resorption followed by bone formation, which is necessary to maintain mineral homeostasis and to repair damaged bone caused by daily physical activity. The cells responsible for this adaptation process are known as osteocytes (the mechanosensing cells that also produce cytokines and hormones), osteoblasts (the bone-forming cells), and osteoclasts (the bone-resorbing cells) [1]. Biochemical markers of bone turnover allow the evaluation of bone remodelling in the entire skeleton. At the local level, bone cell activity is usually assessed by two-dimensional (2D) dynamic histomorphometry using fluorescent labels to indicate newly formed bone. In humans, this is typically performed based on iliac crest biopsies quantifying the rates of bone formation and bone resorption [2]. However, histomorphometry is an invasive and destructive technique limited to a single time point and to a non-load-bearing site that is not necessarily representative of most other skeletal sites [3, 4]. Therefore there is a need for a non-invasive and non-destructive technique for the assessment of local *in vivo* bone remodelling in humans.

Recently, the assessment of bone remodelling has received increased attention using non-invasive and non-destructive *in vivo* micro-computed tomography (micro-CT) in animal experimental studies [5–8] (for a recent review, see Christen and Müller [9]). Waarsing et al. [5] have proposed a three-dimensional (3D) method to analyse bone remodelling using time-lapse *in vivo* micro-CT images. It is based on the image registration of two subsequent scans of the same animal where image voxels present only in the segmented baseline scan are considered bone resorption and voxels present only in the segmented follow-up scan are considered bone formation. Using the same approach, Brouwers et al. [6] studied the effects of parathyroid hormone (PTH) treatment on trabecular and cortical bone in ovariectomised rats over 6 weeks. They found that PTH treatment leads to an increase in trabecular bone mass and that formed bone packets are locally added where it is most beneficial for increasing bone strength. Recently, Schulte et al. [10] used time-lapse *in vivo* micro-CT in mice and showed that local bone formation is associated with high mechanical loading whereas local bone resorption is associated with low mechanical loading. Furthermore, the same group established an approach to directly extract bone formation and bone resorption parameters from time-lapse *in vivo* micro-CT images and therefore a protocol for dynamic bone remodelling parameters [7, 11].

High-resolution peripheral quantitative CT (HR-pQCT) has the potential to characterise the 3D bone microstructure *in vivo* in patients at peripheral sites such as the distal radius and tibia with a nominal isotropic voxel size of 82 or 61 micrometres (for a recent review, see Christen and Müller [9]). Recently we showed that applying similar approaches of image registration as that used in the animal experimental studies indeed enables the identification of bone formation and bone resorption sites during a 2-year follow-up period and that these correlate well with high and low local mechanical loading conditions, respectively [12]. However, unlike the animal experimental studies, where segmented images were subtracted to identify bone remodelling sites, additional image processing in the form of filtering was required to avoid incorrect classification of too many voxels into a bone remodelling site. The settings of this filtering determine the least-detectable local change between follow-up HR-pQCT images. While in the previous study a rather aggressive filtering was applied ensuring the removal of all noise, knowing the limits of the approach would potentially allow use of filtering settings that only remove the noise and not bone remodelling sites. The aim of the present study was therefore to quantify these filtering settings using *ex vivo* reproducibility HR-pQCT images, to determine the least-detectable bone remodelling change using *in vivo* reproducibility HR-pQCT images, and to test whether the approach is capable of capturing the effects of ageing on long-term bone remodelling in postmenopausal women using time-lapse *in vivo* HR-pQCT images.

## Materials and methods

### Subjects

For the *ex vivo* reproducibility HR-pQCT images, scanning was performed on a total of 15 fresh frozen cadaveric forearms (left forearm, male/female: 21-81/45-95 years old, respectively). All forearms were disarticulated at the elbow, thawed and scanned three times within 48 hours. The time lag between each scan was within the same day and scanning was performed by a single operator. None of the donors were from a vulnerable population and all donors or next of kin provided written informed consent that was freely given. The study was approved by an independent Ethics Committee (Comité de Protection des Personnes Sud-Est II).

For the *in vivo* reproducibility HR-pQCT images, image data from a previous study [13] were used. Scanning was performed on a total of 15 healthy individuals (21–47 years old). All of them were scanned in three separate occasions within one month intervals at both the non-dominant radius and tibia. The time lag between two scans varied between one day and one week.

For the time-lapse *in vivo* HR-pQCT images, scans of 9 untreated postmenopausal women (50–80 years old) with no history of previous fracture or bone-related ailments were chosen from the OFELY Cohort [14, 15]. All of them were measured by HR-pQCT at the distal tibia at baseline and after 2, 4, and 6 years follow-up. The study was approved by an independent Ethics Committee and all the involved subjects gave written informed consent before participation.

### HR-pQCT imaging

Imaging was performed in the present and also the previous studies [13–15] using a clinical HR-pQCT system (XtremeCT, Scanco Medical AG, Brüttisellen, Switzerland) providing images with a voxel size of 82 micrometres. The image quality was independently graded by two trained observers and according to the image grading system suggested by the manufacturer. Five different grades were defined from grade 1 (no visible motion artefacts) to grade 5 (severe motion artefacts). The major criteria for degraded quality due to motion were based on the presence and extent of horizontal streaking, disruption of cortical contiguity, and trabecular smearing [16].

### Image registration

Several image registration algorithms have been reported for the processing of time-lapse *in vivo* micro-CT images [5, 17]. Here, we use 3D rigid image registration and an algorithm that maximises image correlation in three different image resolution steps as implemented in the scanner software (Image Processing Language IPL, V5.16/FE-v01.16, Scanco Medical AG, Brüttisellen, Switzerland). The image registration method was applied to the grey-level images (2 bytes per voxel, 110 slices) where the follow-up images were registered individually to the baseline images. This procedure provides a transformation matrix that contains rigid body rotations and translations needed to transform the follow-up image and the associated mask (periosteal contours) to the baseline image. Finally, images were masked with a largest common volume (LCV) mask to exclude any voxels outside the common region. The LCV was determined as the region where the baseline and transformed follow-up masks overlap. The registration process is described in more detail elsewhere [6, 18, 19].

### Image analysis and processing

The density values of the baseline and follow-up images were subtracted voxel-by-voxel within the LCV. The resulting grey-level image therefore represents differences in local densities between follow-up images. These differences can indicate local bone remodelling, but can also be related to artefacts that would be revealed in reproducibility image data such as differences in calibration between scans, errors introduced by interpolating registered images, and noise in the images. To reduce the effects of artefacts on the results, a two-step filter approach was previously developed [12]. In the first step, a global threshold expressed in milligram hydroxyapatite per cubic centimetres (mg HA/cm^3^) is defined and only voxels for which the change in bone density exceeds the positive and negative value of the threshold are classified as sites of bone formation and bone resorption, respectively. The other voxels are classified as unchanged sites. In the second step, starting with the classified voxels from the first step, only those voxels forming consistent clusters of at least 5 voxels are filtered. This step removes most of the noise from the images. Since the choice of the threshold value largely determines the outcome of the approach, several threshold values were tested in the present study. Note that no Laplace-Hamming filtering is performed as recommended by the manufacturer for standard HR-pQCT image segmentation because this filter would not retain the voxel grey-level-density calibration. To visualise the results, bone formation and bone resorption voxels are overlapped with the segmented baseline image resulting in a binary image containing only information inside the LCV indicating unchanged bone (grey) and local bone formation (green) and resorption (magenta). Each image was segmented individually to match the bone volume to total volume as calculated by dividing the mean trabecular density by 1200 mg HA/cm^3^, which is assumed to represent fully mineralised bone [20–23]. This segmentation method for visualising local bone remodelling was chosen to best represent the local bone remodelling as determined by the present two-step filtering approach that is also based on the trabecular bone density.

### Test cases

To quantify the filter setting, the *ex vivo* reproducibility HR-pQCT images were used. The threshold value for filtering was varied from 200 to 275 mg HA/cm^3^ in steps of 25 mg HA/cm^3^ and the number of voxels classified as bone formation and bone resorption were assessed for each case. Differences in voxel values in this study can relate to errors in the reproducibility of the images, calibration errors, and errors in the 3D registration algorithm. No bone remodelling sites should be detected in the case in the reproducibility image data. This test case, therefore, quantifies the real least-detectable bone remodelling with a single scanner at a single time point, which defines that no smaller effects can be detected with the present approach.

To determine the least-detectable *in vivo* bone remodelling, the *in vivo* reproducibility HR-pQCT images were used. Because, in this case, bone remodelling could be falsely determined as a result of motion artefacts, baseline and follow-up images (per pair) were classified into three different groups according to their image quality (no visible motion artefacts, visible motion artefacts, and severe motion artefacts).

To test the sensitivity of the approach for capturing the effects of ageing on long-term bone remodelling in postmenopausal women when using the parameters determined in the earlier studies, the time-lapse *in vivo* HR-pQCT images were used.

### Statistical analysis

From the three-coloured images generated by the present image analysis, volumes defined as resorbed and formed bone (mm^3^) were isolated and expressed as a percentage of the baseline bone volume (BV). For the threshold values ranging from 200 to 275 mg HA/cm^3^, the *ex vivo* least-detectable bone remodelling (mean ± SD, %) was determined by averaging the percentage of resorbed and formed bone over all cadaveric forearms. The *in vivo* least-detectable bone remodelling (mean ± SD, %) was determined for all images and then for each image quality group individually. Finally, age-related bone remodelling (mean ± SD, %) in postmenopausal women was determined independently from image quality and at 2, 4, and 6 years follow-up.

## Results

### Image quality

Image quality was good in the observed cadaveric forearms (all considered as grade 1). Among the 90 *in vivo* reproducibility scans (45 radius and 45 tibia), the image quality ranged from grade 1 to 3 (grade 1: n = 10/25, grade 2: n = 22/17, and grade 3: n = 13/3, for the radius/tibia, respectively). In the time-lapse *in vivo* age-related bone remodelling image data, no scan was excluded and overall scanning had good image quality (grade 1).

### Image registration

A good visual and quantitative agreement (correlation coefficient r>0.99) was obtained for all 3D image registrations. After the registration, the average percentage of the LCV retained for analysis between baseline and follow-up scans was 90.4%, ranging from 77.1% to 95.8%.

### Threshold value

**Fig 1:**
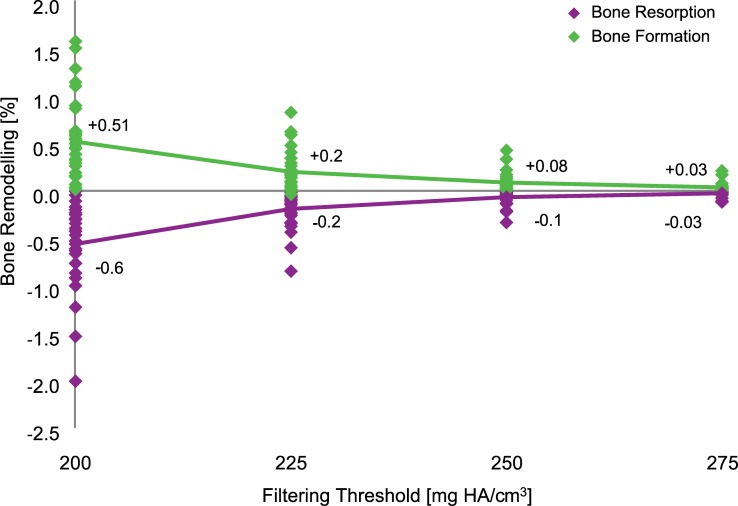
*Ex vivo* bone remodelling for filtering threshold values between 200 and 275 mg HA/cm^3^. The number of voxels considered as bone formation and bone resorption are expressed as the percentage of the number of bone voxels at baseline.

The *ex vivo* local bone remodelling expressed as a percentage of the number of baseline bone voxels ranged from 0.6% to 0.03% for threshold values between 200 and 275 mg HA/cm^3^ (Fig 1). Setting a threshold of 225 mg HA/cm^3^ resulted in volumes of bone formation of 0.2% and bone resorption of 0.2% and therefore, added together, of less than 0.5% of the baseline bone volume. 0.5% can be regarded as an acceptable noise level and thus a threshold of 225 mg HA/cm^3^ is considered here to be the recommended threshold value. It was used for further analysis in the present study.

### Least-detectable local *in vivo* bone remodelling

**Fig 2:**
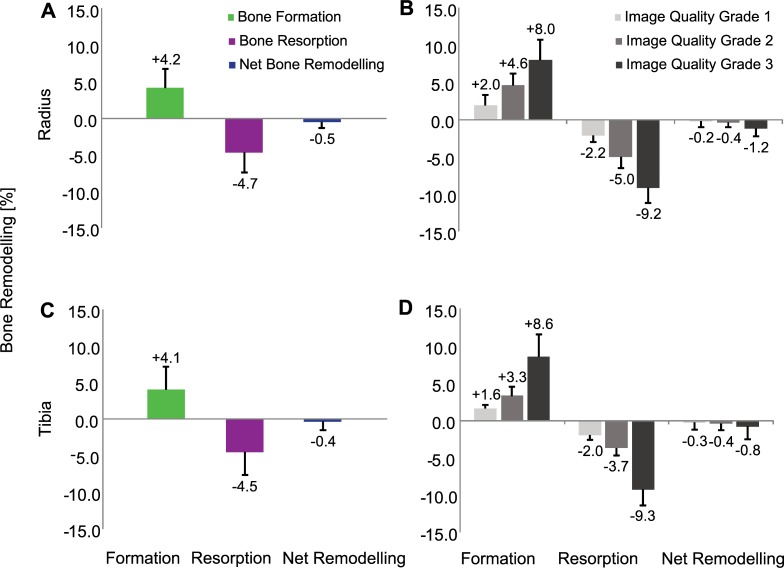
*In vivo* bone formation, bone resorption and net bone remodelling expressed as percentage of the number of baseline bone voxels at the radius (A and B) and tibia (C and D) including all image quality (A and C) as well as per image quality grading (B and D).

Analysing the *in vivo* reproducibility HR-pQCT images of the radius and tibia, bone formation and bone resorption of 4.7 ± 2.7% on average were found (Fig 2A and Fig 2C). This analysis also included low-quality images. In contrast, only analysing high-quality images led to lower bone formation of 2.0 ± 1.0%, bone resorption of 2.2 ± 0.7%, and a net change of 0.2 ± 0.6% on average in the radius and tibia (Fig 2B and Fig 2D).

### Age-related local bone remodelling

**Fig 3:**
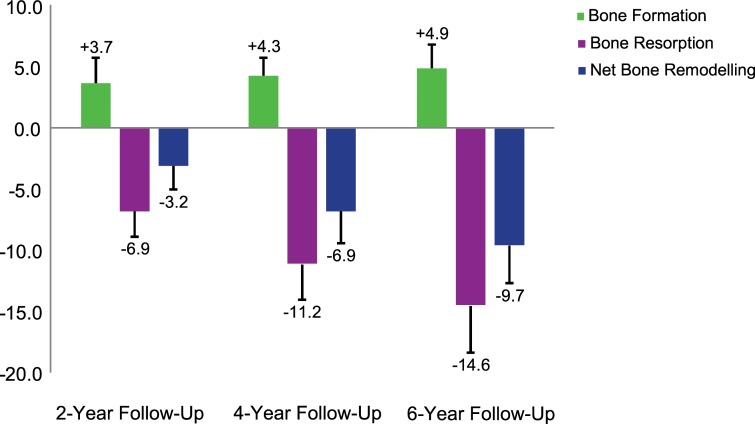
Age-related *in vivo* bone formation, bone resorption and net bone remodelling at the tibia over 2, 4 and 6 years, expressed as percentage of the number of baseline bone voxels number.

**Fig 4:**
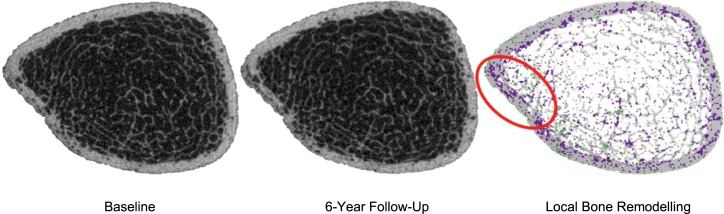
Grey-level cross-sectional images at baseline and 6-year follow-up and resulting local *in vivo* bone remodelling after 6 years. Grey voxels represent common bone; green and magenta coloured voxels represent bone formation and bone resorption, respectively.

Local *in vivo* bone remodelling was further assessed in postmenopausal women at the tibia over 2, 4, and 6 years using the determined threshold value of 225 mg HA/cm^3^. Bone formation ranged on average from 3.7% to 4.9% whereas bone resorption ranged on average from 6.9% to 14.6%, resulting in a net decrease of 3.2%, 6.9% and 9.7% over 2, 4, and 6 years, respectively (Fig 3). In comparison with the bone remodelling assessed within the short-term period of one month, this analysis shows that over 6 years, there seems to be a much higher net decrease (9.7% versus 0.5%). Visualising the local bone remodelling reveals that the cortical structure becomes thinner over time, particularly due to an increase in cortical porosity (Fig 4).

## Discussion

We found that the filtering approach developed here leads to acceptable results: the least-detectable *in vivo* bone formation and bone resorption are 2.0 ± 1.0% and 2.2 ± 0.7%, respectively, and the method is sensitive enough to detect age-related local bone remodelling in postmenopausal women over a 2-year follow-up period. The reproducibility of bone morphometric parameters of single-centre [24, 25] and also multi-centre [26] *in vivo* HR-pQCT is of similar magnitude as our least-detectable *in vivo* bone remodelling and thus acceptable. Please note that the multi-centre error does not play a role if the imaging is always performed at the same centre for each time point, which is usually the case. At the 2-year follow-up, the detected bone formation (3.7%) and bone resorption (-6.9%) exceeded the detection limit by a factor of about 2 or more, suggesting that already at the 1-year follow-up reliable detection of bone formation and bone resorption is possible. Since the age-related local bone remodelling revealed in our study is higher than the reproducibility of bone morphometric parameters of *in vivo* HR-pQCT, our results furthermore indicate that age-related changes were found. A threshold value of 225 mg HA/cm^3^ was chosen as the filter setting because it leads to a small and acceptable total error of less than 0.5% which is in agreement with the reproducibility of bone density of *in vivo* HR-pQCT [24, 25]. The least-detectable local *in vivo* bone remodelling (2% of the baseline volume) is small enough to capture even minor changes in the bone microstructure. However, this is only true for good quality images since the least-detectable local *in vivo* bone remodelling is increased to 4.7% if including lower quality images. Consistent with our findings, Waarsing et al. [27] reported an error in bone volume smaller than 3% using time-lapse *in vivo* micro-CT in animals. They reported that their registration algorithm might cause a shift of about one pixel between positions of bone surfaces when registered scans were overlapped. For *in vivo* HR-pQCT, such a 1-voxel offset would result in considerably larger changes in bone density than the few percent here.

The analysis of age-related local bone remodelling revealed that the rate of bone resorption was much higher than that of bone formation resulting in an average net decrease of about 1.6% per year at the cortical and trabecular level. A comparison of directly extracted volumes between bone formation and bone resorption in each woman revealed that the amount of formed bone is considerably lower than the resorbed one. This implies that, with age, bone remodelling seems to take place by few small formation packets and many large resorption volumes.

Furthermore, visualisation of the three-coloured images at the 2, 4 and 6 year follow-up shows that trabeculae become progressively thinner, perforated, and then removed from the trabecular compartment due to ageing. Dalzell et al. [28] found that bone mineral density declines with age by assessing 132 subjects between 20 and 79 years old using HR-pQCT, which is in line with our results as well as with the findings of the OFELY cohort [29]. However, they found no significant association between trabecular bone parameters and age, which contrasts with our results. Burt et al. [30] determined age-specific centile curves for HR-pQCT parameters in 866 subjects between 16 and 98 years old. In agreement with Dalzell et al. [28], these curves show a pronounced effect of age on bone mineral density but they also show that trabecular parameters are affected by age, particularly in women and after the age of 60. The latter could be an explanation why Dalzell et al. [28] found no association between age and trabecular parameters since they used subjects up to an age of 79 while the age range was larger in Burt et al. [30] and in the present study where, in addition, only female subjects were studied. The cortex seems to be trabecularised by large and coalesced pores leaving cortical remnants that look similar to trabeculae (Fig 4). This is in agreement with the finding of Bala et al. [31], reporting cortical and trabecular fragmentation during aging. We know that the inclusion of cortical remnants as trabeculae lead to an overestimation of trabecular density in old age. Additionally, calculation of porosity [32, 33] in the residual compact cortex without inclusion of the porosity that trabecularised the cortex resulted in a three-fold underestimate of the age-related cortical porosity increase. Our technique could potentially overcome this underestimation and might provide an accurate assessment of cortical and trabecular bone resorption in the original cortical and trabecular compartments allowing a determination of true age-related bone resorption.

It should be noted that our study has some limitations. We used transformed follow-up images, which are slightly less detailed due to the interpolation required for the transformation [34]. Presently, it is not known to what extent such errors contribute to the reproducibility of HR-pQCT in general and the filter setting of our approach in particular. Motion artefacts represent a common finding during *in vivo* HR-pQCT image acquisition, which could result in reproducibility errors and might also affect the image registration. From previous studies, it has been reported that increased subject motion introduces errors resulting in lower bone mineral density [35]. This is why we do not recommend including low quality images (≥ grade 3) while using the present image analysis approach. Finally, a general limitation of the presented method is the current image resolution provided by the HR-pQCT system. Traditionally, dynamic bone parameters could be assessed by 2D histomorphometry (gold standard), even within a short period of time, providing an overview of bone cell activity. Unfortunately, with the current HR-pQCT resolution, this is not possible. In our case, the minimum time interval is dependent on the voxel size, which is 82 micrometres for the first generation of HR-pQCT devices, as used here, or 61 micrometres for the second generation of HR-pQCT devices. Here we only used the first generation HR-pQCT, and thus we do not know if these settings are optimal for the second generation HR-pQCT scanners.

In conclusion, we found that local *in vivo* bone remodelling can be successfully assessed with time-lapse *in vivo* HR-pQCT. The approach is capable of capturing age-related *in vivo* bone remodelling over a 2-year time period indicating that the effects of disorders might also be possible to capture as the related changes would be expected to be larger.

## References

[1] Crockett JC, Rogers MJ, Coxon FP, Hocking LJ, Helfrich MH. Bone remodelling at a glance. J Cell Sci. 2011;124(7):991–8. 21402872 10.1242/jcs.063032

[2] Parfitt AM, Drezner MK, Glorieux FH, Kanis JA, Malluche H, Meunier PJ, et al Bone histomorphometry: standardization of nomenclature, symbols, and units. Report of the ASBMR Histomorphometry Nomenclature Committee. Journal of bone and mineral research: the official journal of the American Society for Bone and Mineral Research. 1987;2(6):595–610. 10.1002/jbmr.5650020617 3455637

[3] Compston JE, Vedi S, Stellon AJ. Inter-observer and intra-observer variation in bone histomorphometry. Calcified tissue international. 1986;38(2):67–70. 3082495 10.1007/BF02556831

[4] Wright CD, Vedi S, Garrahan NJ, Stanton M, Duffy SW, Compston JE. Combined inter-observer and inter-method variation in bone histomorphometry. Bone. 1992;13(3):205–8. 1637566 10.1016/8756-3282(92)90198-6

[5] Waarsing JH, Day JS, van der Linden JC, Ederveen AG, Spanjers C, De Clerck N, et al Detecting and tracking local changes in the tibiae of individual rats: a novel method to analyse longitudinal in vivo micro-CT data. Bone. 2004;34(1):163–9. 14751574 10.1016/j.bone.2003.08.012

[6] Brouwers JE, van Rietbergen B, Huiskes R, Ito K. Effects of PTH treatment on tibial bone of ovariectomized rats assessed by in vivo micro-CT. Osteoporosis international: a journal established as result of cooperation between the European Foundation for Osteoporosis and the National Osteoporosis Foundation of the USA. 2009;20(11):1823–35. 10.1007/s00198-009-0882-5 PMC2765647 19262974

[7] Schulte FA, Lambers FM, Kuhn G, Müller R. In vivo micro-computed tomography allows direct three-dimensional quantification of both bone formation and bone resorption parameters using time-lapsed imaging. Bone. 2011;48(3):433–42. doi: 10.1016/j.bone.2010.10.007 20950723 10.1016/j.bone.2010.10.007

[8] Schulte FA, Lambers FM, Webster DJ, Kuhn G, Muller R. In vivo validation of a computational bone adaptation model using open-loop control and time-lapsed micro-computed tomography. Bone. 2011;49(6):1166–72. doi: 10.1016/j.bone.2011.08.018 21890010 10.1016/j.bone.2011.08.018

[9] Christen P, Müller R. In vivo Visualisation and Quantification of Bone Resorption and Bone Formation from Time-Lapse Imaging. Curr Osteoporos Rep. 2017;15(4):311–7. doi: 10.1007/s11914-017-0372-1 28639146 10.1007/s11914-017-0372-1

[10] Schulte FA, Ruffoni D, Lambers FM, Christen D, Webster DJ, Kuhn G, et al Local Mechanical Stimuli Regulate Bone Formation and Resorption in Mice at the Tissue Level. Plos One. 2013;8(4). 10.1371/journal.pone.0062172 PMC3634859 23637993

[11] Lambers FM, Kuhn G, Schulte FA, Koch K, Müller R. Longitudinal assessment of in vivo bone dynamics in a mouse tail model of postmenopausal osteoporosis. Calcif Tissue Int. 2012;90(2):108–19. doi: 10.1007/s00223-011-9553-6 22159822 10.1007/s00223-011-9553-6

[12] Christen P, Ito K, Ellouz R, Boutroy S, Sornay-Rendu E, Chapurlat RD, et al Bone remodelling in humans is load-driven but not lazy. Nat Commun. 2014;5:5. 10.1038/ncomms5855 25209333

[13] Boutroy S, Bouxsein ML, Munoz F, Delmas PD. In vivo assessment of trabecular bone microarchitecture by high-resolution peripheral quantitative computed tomography. J Clin Endocrinol Metab. 2005;90(12):6508–15. doi: 10.1210/jc.2005-1258 16189253 10.1210/jc.2005-1258

[14] Arlot ME, Sornay-Rendu E, Garnero P, Vey-Marty B, Delmas PD. Apparent pre- and postmenopausal bone loss evaluated by DXA at different skeletal sites in women: the OFELY cohort. Journal of bone and mineral research: the official journal of the American Society for Bone and Mineral Research. 1997;12(4):683–90. 10.1359/jbmr.1997.12.4.683 9101381

[15] Garnero P, Munoz F, Borel O, Sornay-Rendu E, Delmas PD. Vitamin D receptor gene polymorphisms are associated with the risk of fractures in postmenopausal women, independently of bone mineral density. J Clin Endocrinol Metab. 2005;90(8):4829–35. doi: 10.1210/jc.2005-0364 15886235 10.1210/jc.2005-0364

[16] Pialat JB, Burghardt AJ, Sode M, Link TM, Majumdar S. Visual grading of motion induced image degradation in high resolution peripheral computed tomography: impact of image quality on measures of bone density and micro-architecture. Bone. 2012;50(1):111–8. doi: 10.1016/j.bone.2011.10.003 22019605 10.1016/j.bone.2011.10.003

[17] Powell KA, Latson L, Ibiwoye MO, Wolfman A, Grabiner MD, Zborowski M, et al In vivo longitudinal assessment of bone resorption in a fibular osteotomy model using micro-computed tomography. The Iowa orthopaedic journal. 2005;25:123–8. 16089084 PMC1888793

[18] Ellouz R, Chapurlat R, van Rietbergen B, Christen P, Pialat JB, Boutroy S. Challenges in longitudinal measurements with HR-pQCT: Evaluation of a 3D registration method to improve bone microarchitecture and strength measurement reproducibility. Bone. 2014. 10.1016/j.bone.2014.03.001 24614646

[19] Verhulp E, van Rietbergen B, Huiskes R. A three-dimensional digital image correlation technique for strain measurements in microstructures. J Biomech. 2004;37(9):1313–20. doi: 10.1016/j.jbiomech.2003.12.036 15275838 10.1016/j.jbiomech.2003.12.036

[20] Laib A, Hauselmann HJ, Ruegsegger P. In vivo high resolution 3D-QCT of the human forearm. Technol Health Care. 1998;6(5–6):329–37. 10100936

[21] Cohen A, Dempster DW, Muller R, Guo XE, Nickolas TL, Liu XS, et al Assessment of trabecular and cortical architecture and mechanical competence of bone by high-resolution peripheral computed tomography: comparison with transiliac bone biopsy. Osteoporosis Int. 2010;21(2):263–73. 10.1007/s00198-009-0945-7 PMC2908272 19455271

[22] Burghardt AJ, Issever AS, Schwartz AV, Davis KA, Masharani U, Majumdar S, et al High-Resolution Peripheral Quantitative Computed Tomographic Imaging of Cortical and Trabecular Bone Microarchitecture in Patients with Type 2 Diabetes Mellitus. J Clin Endocr Metab. 2010;95(11):5045–55. doi: 10.1210/jc.2010-0226 20719835 10.1210/jc.2010-0226 PMC2968722

[23] Burghardt AJ, Kazakia GJ, Majumdar S. A local adaptive threshold strategy for high resolution peripheral quantitative computed tomography of trabecular bone. Ann Biomed Eng. 2007;35(10):1678–86. doi: 10.1007/s10439-007-9344-4 17602299 10.1007/s10439-007-9344-4

[24] Boutroy S, Bouxsein ML, Munoz F, Delmas PD. In vivo assessment of trabecular bone microarchitecture by high-resolution peripheral quantitative computed tomography. J Clin Endocr Metab. 2005;90(12):6508–15. doi: 10.1210/jc.2005-1258 16189253 10.1210/jc.2005-1258

[25] MacNeil JA, Boyd SK. Improved reproducibility of high-resolution peripheral quantitative computed tomography for measurement of bone quality. Med Eng Phys. 2008;30(6):792–9. doi: 10.1016/j.medengphy.2007.11.003 18164643 10.1016/j.medengphy.2007.11.003

[26] Burghardt AJ, Pialat JB, Kazakia GJ, Boutroy S, Engelke K, Patsch JM, et al Multicenter precision of cortical and trabecular bone quality measures assessed by high-resolution peripheral quantitative computed tomography. J Bone Miner Res. 2013;28(3):524–36. doi: 10.1002/jbmr.1795 23074145 10.1002/jbmr.1795 PMC3577969

[27] MacNeil JA, Boyd SK. Improved reproducibility of high-resolution peripheral quantitative computed tomography for measurement of bone quality. Med Eng Phys. 2008;30(6):792–9. doi: 10.1016/j.medengphy.2007.11.003 18164643 10.1016/j.medengphy.2007.11.003

[28] Dalzell N, Kaptoge S, Morris N, Berthier A, Koller B, Braak L, et al Bone micro-architecture and determinants of strength in the radius and tibia: age-related changes in a population-based study of normal adults measured with high-resolution pQCT. Osteoporosis Int. 2009;20(10):1683–94. 10.1007/s00198-008-0833-6 19152051

[29] Arlot ME, SornayRendu E, Garnero P, VeyMarty B, Delmas PD. Apparent pre- and postmenopausal bone loss evaluated by DXA at different skeletal sites in women: The OFELY cohort. J Bone Miner Res. 1997;12(4):683–90. doi: 10.1359/jbmr.1997.12.4.683 9101381 10.1359/jbmr.1997.12.4.683

[30] Burt LA, Liang ZY, Sajobi TT, Hanley DA, Boyd SK. Sex- and Site-Specific Normative Data Curves for HR-pQCT. J Bone Miner Res. 2016;31(11):2041–7. doi: 10.1002/jbmr.2873 27192388 10.1002/jbmr.2873

[31] Bala Y, Bui QM, Wang XF, Iuliano S, Wang QJ, Ghasem-Zadeh A, et al Trabecular and Cortical Microstructure and Fragility of the Distal Radius in Women. J Bone Miner Res. 2015;30(4):621–9. doi: 10.1002/jbmr.2388 25327362 10.1002/jbmr.2388

[32] Zebaze R, Ghasem-Zadeh A, Mbala A, Seeman E. A new method of segmentation of compact-appearing, transitional and trabecular compartments and quantification of cortical porosity from high resolution peripheral quantitative computed tomographic images. Bone. 2013;54(1):8–20. doi: 10.1016/j.bone.2013.01.007 23334082 10.1016/j.bone.2013.01.007

[33] Zebaze RMD, Ghasem-Zadeh A, Bohte A, Iuliano-Burns S, Mirams M, Price RI, et al Intracortical remodelling and porosity in the distal radius and post-mortem femurs of women: a cross-sectional study. Lancet. 2010;375(9727):1729–36. doi: 10.1016/S0140-6736(10)60320-0 20472174 10.1016/S0140-6736(10)60320-0

[34] Schulte FA, Lambers FM, Mueller TL, Stauber M, Müller R. Image interpolation allows accurate quantitative bone morphometry in registered micro-computed tomography scans. Comput Method Biomec. 2014;17(5):539–48. 10.1080/10255842.2012.699526 22746535

[35] Pauchard Y, Ayres FJ, Boyd SK. Automated quantification of three-dimensional subject motion to monitor image quality in high-resolution peripheral quantitative computed tomography. Phys Med Biol.56(20):6523–43. doi: 10.1088/0031-9155/56/20/001 21937776 10.1088/0031-9155/56/20/001

